# Drifting States and Synchronization Induced Chaos in Autonomous Networks of Excitable Neurons

**DOI:** 10.3389/fncom.2016.00098

**Published:** 2016-09-21

**Authors:** Rodrigo Echeveste, Claudius Gros

**Affiliations:** Institute for Theoretical Physics, Goethe-Universität FrankfurtFrankfurt, Germany

**Keywords:** synchronization, chaos, neural network, integrate-and-fire neuron, excitatory neurons, phase diagrams

## Abstract

The study of balanced networks of excitatory and inhibitory neurons has led to several open questions. On the one hand it is yet unclear whether the asynchronous state observed in the brain is autonomously generated, or if it results from the interplay between external drivings and internal dynamics. It is also not known, which kind of network variabilities will lead to irregular spiking and which to synchronous firing states. Here we show how isolated networks of purely excitatory neurons generically show asynchronous firing whenever a minimal level of structural variability is present together with a refractory period. Our autonomous networks are composed of excitable units, in the form of leaky integrators spiking only in response to driving currents, remaining otherwise quiet. For a non-uniform network, composed exclusively of excitatory neurons, we find a rich repertoire of self-induced dynamical states. We show in particular that asynchronous drifting states may be stabilized in purely excitatory networks whenever a refractory period is present. Other states found are either fully synchronized or mixed, containing both drifting and synchronized components. The individual neurons considered are excitable and hence do not dispose of intrinsic natural firing frequencies. An effective network-wide distribution of natural frequencies is however generated autonomously through self-consistent feedback loops. The asynchronous drifting state is, additionally, amenable to an analytic solution. We find two types of asynchronous activity, with the individual neurons spiking regularly in the pure drifting state, albeit with a continuous distribution of firing frequencies. The activity of the drifting component, however, becomes irregular in the mixed state, due to the periodic driving of the synchronized component. We propose a new tool for the study of chaos in spiking neural networks, which consists of an analysis of the time series of pairs of consecutive interspike intervals. In this space, we show that a strange attractor with a fractal dimension of about 1.8 is formed in the mentioned mixed state.

## 1. Introduction

The study of collective synchronization has attracted the attention of researchers across fields for now over half a century (Winfree, 1967; Kuramoto, 1975; Peskin, 1975; Buck, 1988; Pikovsky and Rosenblum, 2015). Kuramoto's exactly solvable mean field model of coupled limit-cycles (Kuramoto, 1975), formulated originally by Winfree (1967), has helped in this context to establish the link between the distribution of natural frequencies and the degree of synchronization (Gros, 2010). Moreover, the functional simplicity of this model, and other extensions, has permitted to analytically study the collective response of the system to external perturbations in the form of phase resets (Levnajić and Pikovsky, 2010). Networks of phase coupled oscillators may show, in addition, attracting states corresponding to limit cycles, heteroclinic networks, and chaotic phases (Ashwin et al., 2007; Dörfler and Bullo, 2014), with full, partial, or clustered synchrony (Golomb et al., 1992), or asynchronous behavior (Abbott and van Vreeswijk, 1993)

Different degrees of collective synchronization may occur also in networks of elements emitting signals not continuously, such as limit-cycle oscillators, but via short-lived pulses (Mirollo and Strogatz, 1990; Abbott and van Vreeswijk, 1993; Strogatz and Stewart, 1993). Networks of pacemaker cells in the heart (Peskin, 1975), for instance, synchronize with high precision, acting together as a robust macroscopic oscillator. Other well-known examples are the simultaneous flashing of extended populations of southeast Asian fireflies (Hanson, 1978; Buck, 1988) and the neuronal oscillations of cortical networks (Buzsáki and Draguhn, 2004). In particular, the study of synchronization in the brain is of particular relevance for the understanding of epileptic states, or seizures (Velazquez et al., 2007).

The individual elements are usually modeled in this context as integrate and fire units (Kuramoto, 1991; Izhikevich, 1999), where the evolution (in between pulses, flashes, or spikes) of a continuous internal state variable *V* is governed by an equation of the type:

(1) $$\tau \dot{V}\;=\;f(V)+I.$$

Here τ is the characteristic relaxation timescale of *V*, with *f* representing the intrinsic dynamics of the unit, and *I* the overall input (both from other units and from external stimuli). Whenever *V* reaches a threshold value *V*_θ_, a pulse is emitted (the only information carried to other units) and the internal variable is reset to *V*_*rest*_.

These units are usually classified either as oscillators or as excitable units, depending on their intrinsic dynamics. The unit will fire periodically even in the absence of input when *f*(*V*) > 0 (∀*V* ≤ *V*_θ_). Units of this kind are denoted *pulse-coupled oscillators*. The unit is, on the other hand, an *excitable unit*, if an additional input is required to induce firing.

A natural frequency given by the inverse integration time of the autonomous dynamics exist in the case of pulse-coupled oscillators. There is hence a preexisting, albeit discontinuous limit cycle, which is then perturbed by external inputs. One can hence use phase coupling methods to study networks of pulse coupled oscillators (Mirollo and Strogatz, 1990; Kuramoto, 1991; Izhikevich, 1999), by establishing a map between the internal state variable *V* and a periodic phase ϕ given by the state of the unit within its limit cycle. From this point of view systems of pulse-coupled units share many properties with sets of coupled Kuramoto-like oscillators (Kuramoto, 1975), albeit with generally more complex coupling functions (Izhikevich, 1999). For reviews and examples of synchronization in populations of coupled oscillators see Strogatz (2000) and Dörfler and Bullo (2014).

These assumptions break down for networks of coupled excitable units as the ones here described. In this case the units will remain silent without inputs from other elements of the system and there are no preexisting limit cycles and consequently also no preexisting natural frequencies (unlike *rotators* (Sonnenschein et al., 2014), which are defined in terms of a periodic phase variable, and a count with a natural frequency). The firing rate depends hence exclusively on the amount of input received. The overall system activity will therefore forcefully either die out or be sustained collectively in a self-organized fashion (Gros, 2010). The respectively generated spiking frequencies for a given mean network activity could be considered in this context as self-generated natural frequencies.

The study of pulse coupled excitable units is of particular relevance within the neurosciences, where neurons are often modeled as spike emitting units that continuously integrate the input they receive from other cells (Burkitt, 2006). The proposal (Shadlen and Newsome, 1994; Amit and Brunel, 1997), and later the empirical observation that excitatory and inhibitory inputs to cortical neurons are closely matched in time (Sanchez-Vives and McCormick, 2000; Haider et al., 2006), has led researchers to focus on dynamical states (asynchronous states in particular) in networks characterized by a balance between excitation and inhibition (Abbott and van Vreeswijk, 1993; van Vreeswijk and Sompolinsky, 1996; Hansel and Mato, 2001; Vogels and Abbott, 2005; Kumar et al., 2008; Stefanescu and Jirsa, 2008). This balance (E/I balance) is generally presumped to be an essential condition for the stability of states showing irregular spiking, such as the one arising in balanced networks of integrate and fire neurons (Brunel, 2000). The type of connectivity usually employed in network studies however, is either global, or local consisting of either repeated patterns, or random connections drawn from identical distributions (Kuramoto and Battogtokh, 2002; Abrams and Strogatz, 2004; Ashwin et al., 2007; Alonso and Mindlin, 2011). Our results show, however, that only a minimal level of structural variability is necessary for excitatory networks to display wide varieties of dynamical states, including stable autonomous irregular spiking. We believe that these studies are not only interesting on their own because of the richness of dynamical states, but also provide valuable insight into the role of inhibition.

Alternatively, one could have built networks of excitatory neurons with high variability in the connection parameters, reproducing realistic connectivity distributions, such as those found in the brain. The large number of parameters involved would make it however difficult to fully characterize the system from a dynamical systems point of view, the approach taken here. An exhaustive phase-space study would also become intractable. We did hence restrict ourselves in the present work to a scenario of minimal variability, as given by a network of globally coupled excitatory neurons, where the coupling strength of each neuron to the mean field is non-uniform. Our key result is that stable irregular spiking states emerge even when only a minimal level of variability is present at a network level.

Another point we would like to stress here is that asynchronous firing states may be stabilized in the absence of external inputs. In the case here studied, there is an additional “difficulty” to the problem, in the sense that the pulse-coupled units considered are in excitable states, remaining quiet without sufficient drive from the other units in the network. The observed sustained asynchronous activity is hence self-organized.

We characterize how the features of the network dynamics depend on the coupling properties of the network and, in particular, we explore the possibility of chaos in the here studied case of excitable units, when partial synchrony is present, since this link has already been established in the case of coupled oscillators with a distribution of natural frequencies (Miritello et al., 2009), while other studies had also shown how stable chaos emerges in inhibitory networks of homogeneous connection statistics (Angulo-Garcia and Torcini, 2014).

## 2. The model

In the current work we study the properties of the self-induced stationary dynamical states in autonomous networks of excitable integrate-and-fire neurons. The neurons considered are characterized by a continuous state variable *V* (as in Equation 1), representing the membrane potential, and a discrete state variable *y* that indicates whether the neuron fires a spike (*y* = 1) or not (*y* = 0) at a particular point in time. More precisely, we will work here with a conductance based (COBA) integrate-and-fire (IF) model as employed in Vogels and Abbott (2005) (here however without inhibitory neurons), in which the evolution of each neuron *i* in the system is described by:

(2) $$\tau {\dot{V}}_{i}\;=\;\left(V_{rest}-V_{i}\right)\;+\;g_{i}\left(E_{ex}-V_{i}\right),$$

where *E*_*ex*_ = 0 mV represents the excitatory reversal potential and τ = 20 ms is the membrane time constant. Whenever the membrane potential reaches the threshold *V*_θ_ = −50 mV, the discrete state of the neuron is set to *y*_*i*_ = 1 for the duration of the spike. The voltage is reset, in addition, to its resting value of *V*_*rest*_ = −60 mV, where it remains fixed for a refractory period of *t*_*ref*_ = 5 ms. Equation (2) is not computed during the refractory period. Except for the times of spike occurrences, the discrete state of the neuron remains *y*_*i*_ = 0 (no spike).

The conductance *g*_*i*_ in Equation (2) integrates the influence of the time series of presynaptic spikes, decaying on the other side in absence of inputs:

(3) $$\tau_{ex}\;{\dot{g}}_{i}\;=\;-g_{i},$$

where τ_*ex*_ = 5 ms is the conductance time constant. Incoming spikes from the *N* − 1 other neurons produce an increase of the conductance *g_i_* → *g_i_* + Δ*g_i_*, with:

(4) $$\Delta g_{i}\;=\;\frac{K_{i}}{N-1}\;\sum_{j\neq i}w_{ij}y_{j}.$$

Here the synaptic weights *w_ij_* represent the intensity of the connection between the presynaptic neuron *j* and the postsynaptic neuron *i*. We will generally employ normalized synaptic matrices with $\sum_{j}w_{ij}/(N-1)=1$. In this way we can scale the overall strength of the incoming connections via *K_i_*, retaining at the same time the structure of the connectivity matrix.

### 2.1. Global couplings

Different connectivity structures are usually employed in the study of coupled oscillators, ranging from purely local rules to global couplings (Kuramoto and Battogtokh, 2002; Abrams and Strogatz, 2004; Ashwin et al., 2007; Alonso and Mindlin, 2011). We start here by employing a global coupling structure, where each neuron is coupled to the overall firing activity of the system:

(5) $$w_{ij}=1\;\forall \;i\neq j,\;w_{ii}=0,$$

which corresponds to a uniform connectivity matrix without self coupling. All couplings are excitatory. The update rule (Equation 4) for the conductance upon presynaptic spiking then take the form:

(6) $$\Delta g_{i}\;=\;K_{i}\frac{\sum_{j\neq i}y_{j}}{N-1}\;=\;K_{i}\bar{r},\;\bar{r}=\frac{\sum_{j\neq i}y_{j}}{N-1},$$

where *r* = *r*(*t*) represents the time-dependent mean field of the network, viz the average over all firing activities. *r* is hence equivalent to the mean field present in the Kuramoto model (Kuramoto, 1975), resulting in a global coupling function as an aggregation of local couplings. With our choice (Equation 5) for the coupling matrix the individual excitable units may be viewed, whenever the mean field *r* is strong enough, as oscillators emitting periodic spikes with an “effective” natural frequency determined by the afferent coupling strength *K_i_*. The resulting neural activities determine in turn the mean field *r*(*t*).

### 2.2. Coupling strength distribution

We are interested in studying networks with non-uniform *K_i_*, We mostly consider here the case of equidistant *K_i_*, defined by:

(7) $$K_{i}\;=\;\bar{K}-\Delta K+\frac{2\Delta K}{N-1}\left(i-1\right),\;i=1,\ldots ,N$$

for the *N* neurons, where *K* represents the mean scaling parameter, and Δ*K*, the maximal distance to the mean. It is possible, alternatively, to use a flat distribution with the *K_i_* drawn from an interval [*K* − Δ*K*, *K* + Δ*K*] around the mean *K*. For large systems there is no discernible difference, as we have tested, between using equidistant afferent coupling strengths *K_i_* and drawing them randomly from a flat distribution.

## 3. Results

Several aspects of our model, in particular the asynchronous drifting state, can be investigated analytically as a consequence of the global coupling structure (Equation 5), as shown in Section 3.1. All further results are obtained from numerical simulations, for which, if not otherwise stated, a timestep of 0.01 ms has been used. We have also set the spike duration to one time-step, although these two parameters can be modified separately if desired, with our results not depending on the choice of the time-step, while the spike width does introduce minor quantitative changes to the results, as later discussed.

### 3.1. Stationary mean-field solution for the drifting state

As a first approach we compute the response of a neuron with coupling constant *K_i_* to a stationary mean field *r*, as defined by Equation (6), representing the average firing rate of spikes (per second) of the network. This is actually the situation present in the asynchronous drifting state, for which the firing rates of the individual units are incommensurate. With *r* being constant we can combine the update rules (3) and (4) for the conductances *g_i_* to

(8) $$\tau_{ex}\;{\dot{g}}_{i}\;=\;-g_{i}+\tau_{ex}K_{i}\bar{r},\;g_{i}^{*}\;=\;\tau_{ex}K_{i}\bar{r},$$

where we have denoted with ${\text{g}}_{i}^{*}$ the steady-state conductance. With the individual conductance becoming a constant we may also integrate the evolution Equation (2) for the membrane potential,

(9) $$\tau {\dot{V}}_{i}\;=\;\left(V_{rest}-V_{i}\right)+\tau_{ex}K_{i}\bar{r}\left(E_{ex}-V_{i}\right),$$

obtaining the time ${\text{t}}_{i}^{*}$ it takes for the membrane potential *V_i_* to reach the threshold *V*_θ_, when starting from the resting potential *V*_*rest*_:

(10) $$t_{i}^{*}\;=\;-\frac{1}{B_{i}}log\left(\frac{B_{i}V_{\theta }-A_{i}}{B_{i}V_{rest}-A_{i}}\right),$$

with:

(11) $$A_{i}\;=\;\frac{V_{rest}+\tau_{ex}K_{i}\bar{r}E_{ex}}{\tau },\;B_{i}\;=\;\frac{1+\tau_{ex}K_{i}\bar{r}}{\tau }.$$

We note, that the threshold potential *V*_θ_ is only reached, if *dV_i_*/*dt* > 0 for all *V_i_* ≤ *V*_θ_. For the ${\text{t}}_{i}^{*}$ to be finite we hence have (from Equation 9)

(12) $$K_{i}\bar{r}>\frac{1}{\tau_{ex}}\left(\frac{V_{\theta }-V_{rest}}{E_{ex}-V_{\theta }}\right).$$

The spiking frequency is *r*_*r*_ = ${\text{T}}_{i}^{-1}$, with the intervals *T_i_* between consecutive spikes given by *T*_*i*_ = ${\text{t}}_{i}^{*}$ + *t_ref_*, when (Equation 12) is satisfied. Otherwise the neuron does not fire. The mean field *r* is defined as the average firing frequency

(13) $$\bar{r}\;=\;\langle r_{i}\rangle \;=\;\langle \frac{1}{t_{i}^{*}+t_{ref}}\rangle$$

of the neurons. Equations (10) and (13) describe the asynchronous drifting state in the thermodynamic limit *N* → ∞. We denote this self-consistency condition for *r* the stationary mean-field (SMF) solution.

### 3.2. Numerical simulations

We studied our model, as defined by Equations (2) and (3), numerically for networks with typically *N* = 100 neurons, a uniform coupling matrix (see Equation 5) and coupling parameters *K* and Δ*K* given by Equation (7). We did not find qualitative changes when scaling the size of the network up to *N* = 400 for testing purposes (and neither with down-scaling), see **Figure 7**. Random initial conditions where used. The network-wide distribution of firing rates is computed after the system settles to a dynamical equilibrium.

**Figure 1:**
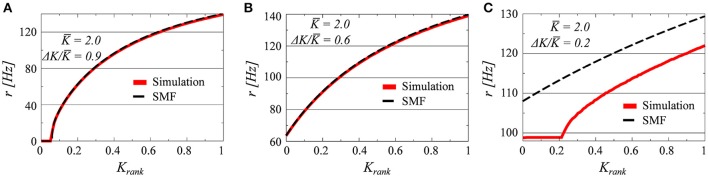
**The firing rates of all *i* = 1, …, *N* neurons, as a function of the relative rank *K*_*rank*_ = *i*/*N* of the individual neurons (*N* = 100)**. The coupling matrix is uniform (see Equation 5) and the afferent coupling strength *K*_*i*_ uniformly distributed between *K* ± Δ*K*; with *K* = 2.0 and Δ*K*/*K* = 0.9/0.6/0.2 **(A–C)**. The full red lines denote the results obtained by solving numerically Equations (2) and (3), and the dashed lines the stationary mean field solution (SMF, Equation 13).

Three examples, for *K* = 2.0 and Δ*K*/*K* = 0.9, 0.6, and 0.2, of firing-rate distributions are presented in Figure 1 in comparison with the analytic results obtained from the stationary mean field approach (*SMF*), as given by Equation (12). The presence or absence of synchrony is directly visible. In all of the three parameter settings presented in Figure 1 there is a drifting component, characterized by a set of neurons with a continuum of frequencies. These neurons fire asynchronously, generating a constant contribution to the collective mean field.

The plateau present in the case Δ*K*/*K* = 0.2, corresponds, on the other hand, to a set of neurons firing with identical frequencies and hence synchronously. Neurons firing synchronously will do so however with finite pairwise phase lags, with the reason being the modulation of the common mean field *r* through the distinct afferent coupling strengths *K_i_*. We note that the stationary mean-field theory (Equation 12) holds, as expected, for drifting states, but not when synchronized clusters of neurons are present.

**Figure 2:**
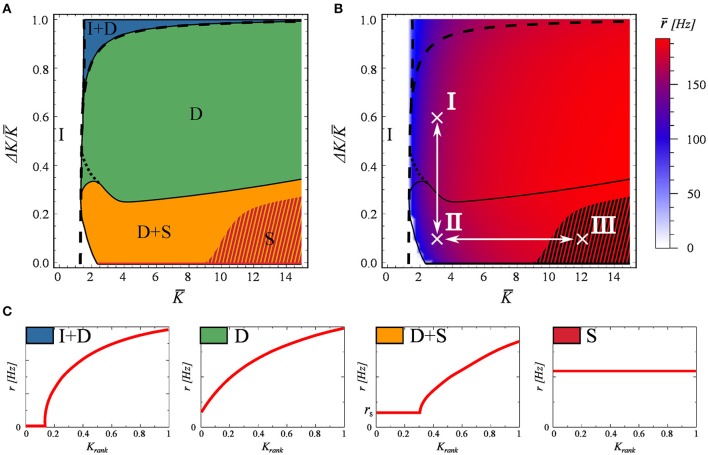
**The phase diagram, as obtained for a network of *N* = 100 neurons evolving according to Equations (2) and (3)**. The network matrix is flat, see Equation (5). Full and partially inactive (I), drifting (D), and synchronized states (S) are found as a function of the coupling parameters *K* and Δ*K* (Equation 7). **(A)** The dashed lines represent the phase transition lines as predicted by the stationary mean field approximation (Equation 13). The shaded region indicates the coexistence of attracting states S and S+D. **(B)** The average firing rate of the network. In black the phase boundaries and in white the two adiabatic paths used in Figure 3. **(C)** Examples of the four active dynamical states found. As in Figure 1.

In Figure 2 we systematically explore the phase space as a function of *K* and Δ*K*. For labeling the distinct phases we use the notation

**Table:** 

I	:	inactive,
I+D	:	partially inactive and drifting,
D	:	fully drifting (asynchronous),
D+S	:	mixed, containing both drifting and synchronized components, and
S	:	fully synchronized.

Examples of the rate distributions present in the individual phases are presented in Figure 2C.

The phase diagram is presented in Figure 2A. The activity dies out for a low mean connectivity strength *K*, but not for larger *K*. Partial synchronization is present when both *K* and the variance Δ*K* are small, taking over completely for larger values of *K* and small Δ*K*. The phase space is otherwise dominated by a fully drifting state. The network average *r* of the neural firing rates, given in Figure 2B, drops only close to the transition to the inactive state I, showing otherwise no discernible features at the phase boundaries.

The dashed lines in Figures 2A,B represent the transitions between the inactive state I and active state I+D, and between states I+D and D, as predicted by the stationary mean field approximation (Equation 13), which becomes exact in the thermodynamic limit. The shaded region in these plots indicates the co-existence of attracting states S and S+D. As a note, we found that the location of this shaded region depends on the spike width, shifting to higher *K* values for narrower spikes. While real spikes in neurons have a finite width, we note from a dynamical systems point of view, that this region would most likely vanish in the limit of delta spikes.

For a stable (non-trivial) attractor to arise in a network composed only of excitatory neurons, some limitation mechanism needs to be at play. Otherwise one observes a bifurcation phenomenon, similar to that of branching problems, in which only a critical network in the thermodynamic limit could be stable (Gros, 2010). In this case, the limiting factor is the refractory period. Refractoriness prevents neurons from firing continuously, and prevents the system activity from exploding. Interestingly, this does not mean that the neurons will fire at the maximal rate of 1/*t_ref_* which would correspond in this case to 200Hz. The existence of this refractory period allows for self-organized states with frequencies even well bellow this limit, as seen in Figure 2B. We have tested these claims numerically by setting *t_ref_* = 0, observing that the neural activity either dies out or the neurons fire continuously.

In order to study the phase transitions between states D and D+S and between D+S and S, we will resort in the following section to adiabatic paths in phase space crossing these lines.

#### 3.2.1. Adiabatic parameter evolution

**Figure 3:**
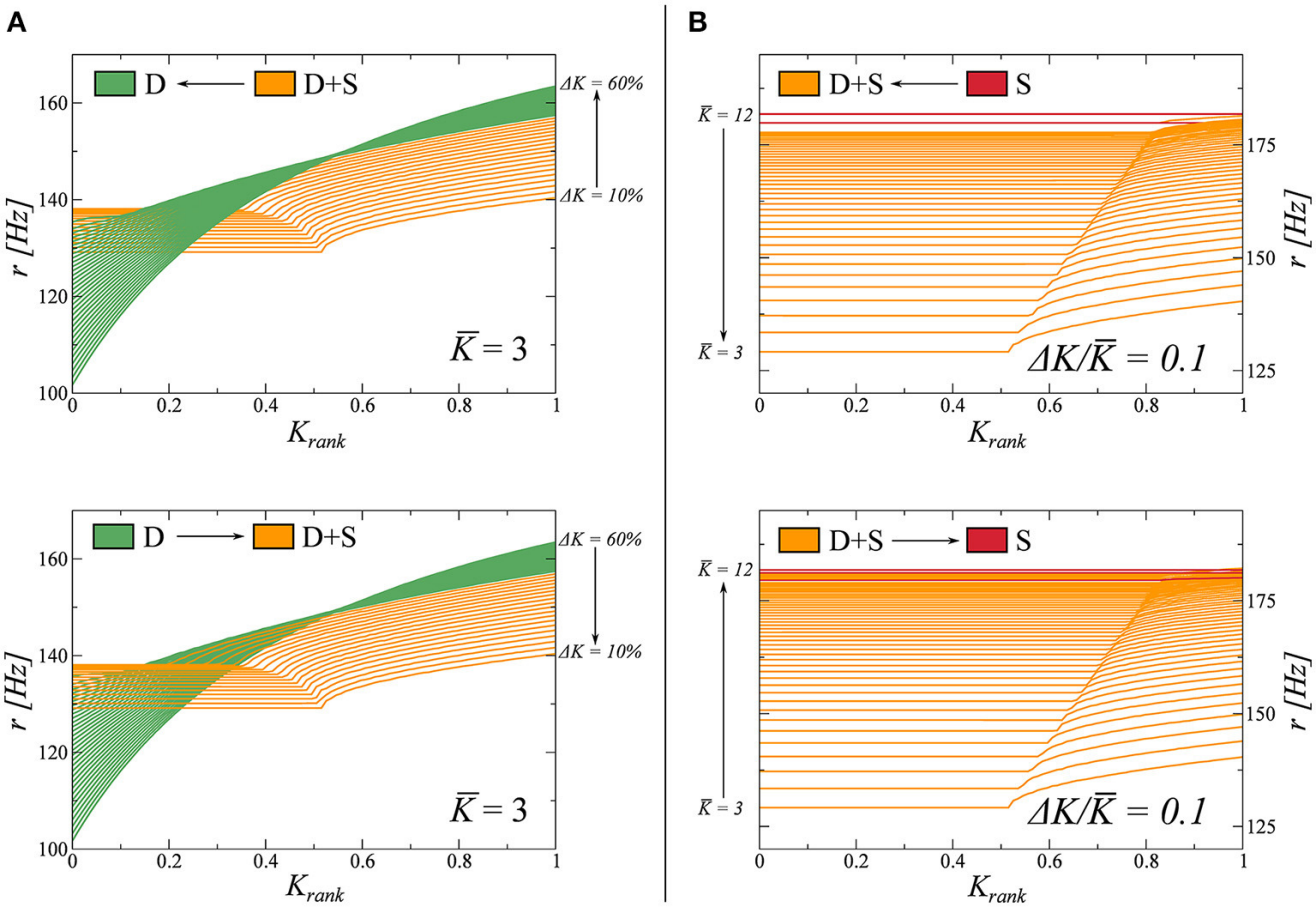
**Study of the transitions between fully drifting *D* and partially drifting and synchronized D+S phase (left panels), and between D+S and the fully synchronized *S* state (right panels)**. For the full phase diagram see Figure 2. The coupling parameters *K* and Δ*K* (Equation 7), are modified on times scales much slower than the intrinsic dynamics. For the two adiabatic paths considered, each crossing a phase transition line, the evolution of the firing rate distribution is computed in several windows and shown. **(A)** *K* = 3.0 is kept constant and *K* varied between 0.1 and 0.6 (from *D* to D+S, indicated as I ↔II in this figure). **(B)** Δ*K*/*K* = 0.1 is kept constant, varying *K* between 3 and 12 (from D+S to *S*, indicated as II ↔III in this figure).

Here we study the nature of the phase transitions between different dynamical states in Figure 2. To do so, we resort to adiabatic trajectories in phase space, crossing these lines. Beginning in a given phase we modify the coupling parameters *K* and Δ*K* (Equation 7), on a timescale much slower than that of the network dynamics. Along these trajectories, we then freeze the system in a number of windows in which we compute the rate distribution as a function of the *K_rank_* (see Figure 1). During these observation windows the parameters do not change. In this way, we can follow how the rate distribution varies across the observed phase transitions. The results are presented in Figure 3.

We observe that the emergence of synchronized clusters, the transition D → (D+S), is completely reversible. We believe this transition to be of second order and that the small discontinuity in the respective firing rate distributions observed in Figure 3A are due to finite-size effects. The time to reach the stationary state diverges, additionally, close to the transition, making it difficult to resolve the locus with high accuracy.

The disappearance of a subset of drifting neurons, the transition S → (D+S) is, on the other hand, not reversible. In this case, when *K* is reduced, the system tends to get stuck in metastable attractors in the *S* phase, producing irregular jumps in the rate distributions. Furthermore, when we increase *K*, we observe that the system jumps back and forth between states D+S and *S* in the vicinity of the phase transition, indicating that both states may coexist as metastable attractors close to the transition. We note that a similar metastability has been observed in partially synchronized phase of the Kuramoto model (Miritello et al., 2009).

#### 3.2.2. Time structure

**Figure 4:**
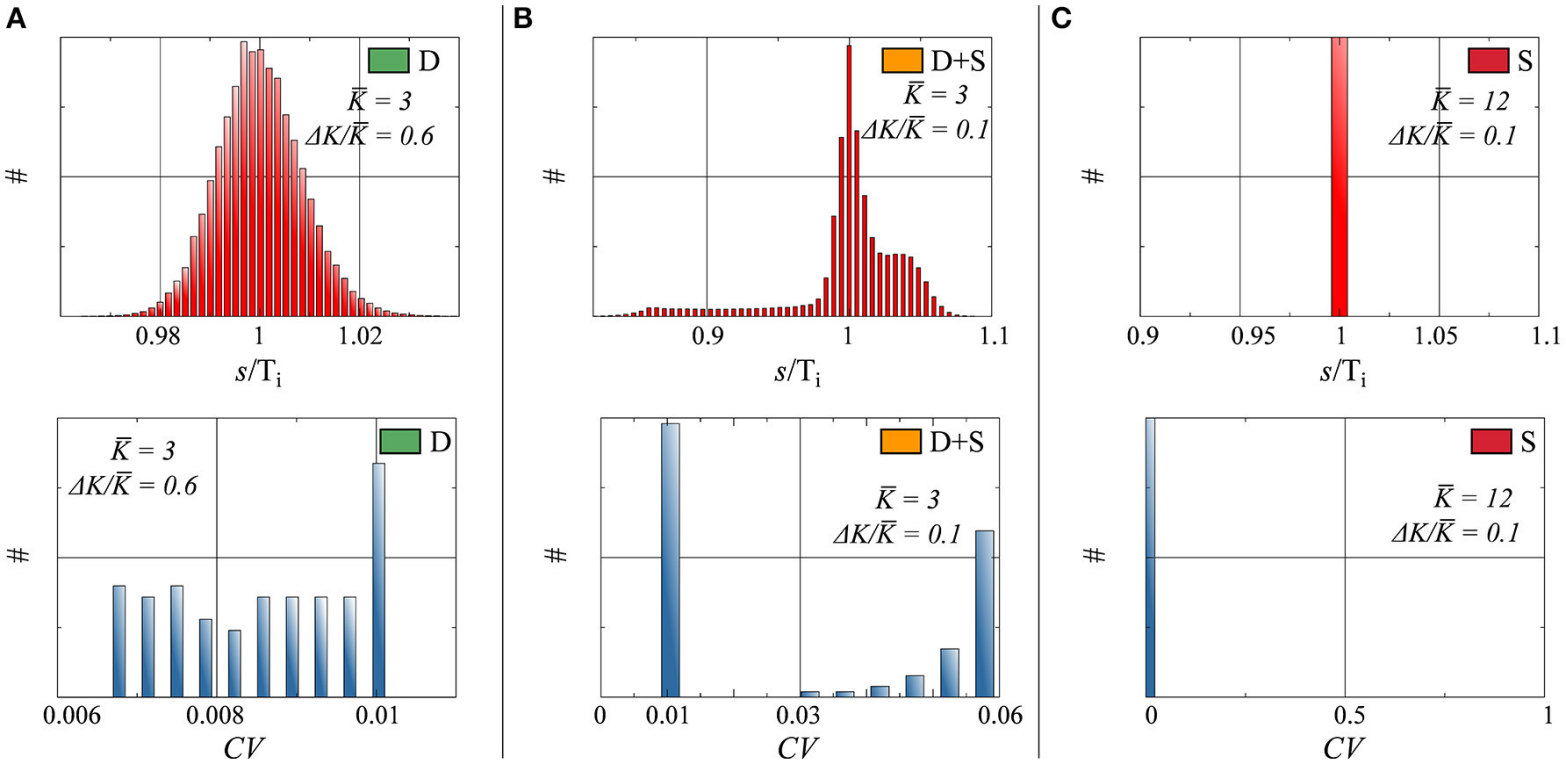
**Top: Histograms of interspike interval (ISI), denoted as s, normalized by the average period, for three parameter configurations**. Bottom: Histograms of the coefficient of variation *CV*, as defined by Equation (14). Parameters (Equation 7) and state (as defined in Figure 2), for both Top and Bottom: **(A)** *K* = 3.0, Δ*K*/*K* = 0.6, state: D. **(B)** *K* = 3.0, Δ*K*/*K* = 0.1, state: D+S. **(C)** *K* = 12.0, Δ*K*/*K* = 0.1, state: S.

In networks of spiking neurons, it is essential to characterize not only the rate distribution of the system, but also the neurons' interspike-time statistics (Perkel et al., 1967a,b; Chacron et al., 2004; Lindner, 2004; Farkhooi et al., 2009). In this case, we have computed the distribution *p_i_*(*s*) of the interspike intervals *s* (*ISI*) of the individual neurons respectively for full and partial drifting and synchronized states. The distribution of inter-spike intervals in Figure 4 shows the network average of the *p_i_*(*s*), normalized individually with respect to the average *T_i_* = ∫ *s p_i_*(*s*)*ds* spiking intervals.

- D : The input received by a given neuron *i* tends to a constant, as discussed in Section 3.1, in the thermodynamic limit *N* → ∞. The small but finite width of the ISI for the fully drifting state D evident in Figure 4 is hence a finite-size effect.
- D+S : The input received for drifting neuron *i* in a state where other neurons form a synchronized subcluster is intrinsically periodic in time and the resulting *p_i_*(*s*) non-trivial, as evident in Figure 4.
- S : *p_i_*(*s*) is a delta function for all neurons in the fully synchronized state, with identical inter-spike intervals *T_i_*.

As a frequently used measure of the regularity of a time distribution we have included in Figure 4 the coefficient of variation (*CV*),

(14) $$CV_{i}=\frac{\sigma_{i}}{T_{i}},\text{​ ​}T_{i}=\int sp_{i}(s)ds,\text{​ ​}\sigma_{i}^{2}=\int {\left(s-T_{i}\right)}^{2}p_{i}(s)ds.$$

Of interest here are the finite *CV* s of the drifting units in the D+S state, which are considerably larger than the *CV* s of the drifting neurons when no synchronized component is present. This phenomenon is a consequence of the interplay between the periodic driving of the drifting neurons by the synchronized subcluster in the D+S state, where the driving frequency will in general be in mismatch with the effective, self-organized natural frequency of the drifting neurons. The firing of a drifting neuron is hence irregular in the mixed D+S state, becoming however regular in the absence of synchronized drivings.

#### 3.2.3. Self induced chaos

The high variability of the spiking intervals observed in the mixed state, as presented in Figure 4, indicates that the firing may have a chaotic component in the mixed state and hence positive Lyapunov exponents (Gros, 2010).

**Figure 5:**
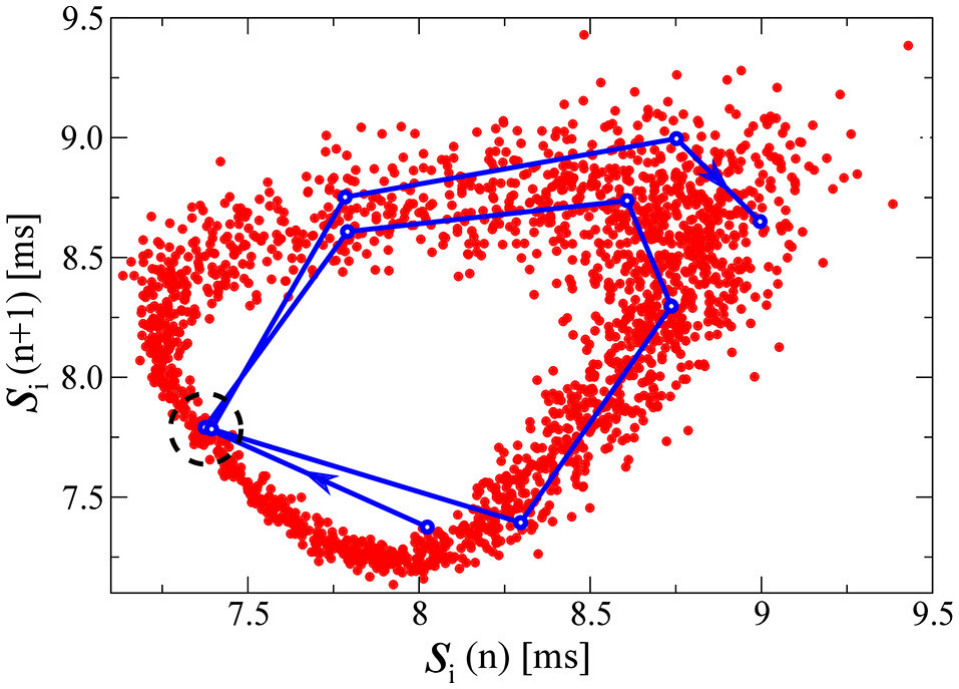
**Pairs of consecutive interspike intervals s plotted against each other, for one of the drifting neurons in the D+S state, corresponding to the third example of Figure 1 (*K* = 2.0 and Δ*K*/*K* = 0.2)**. The plots are qualitatively similar for all drifting neurons in this state. The qualitative features of the plot are the same for any of the drifting neurons in this state. In red, each point represents a pair [*s_i_*(*n*), *s_i_*(*n* + 1)] where n denotes the spike number. In blue, we follow a representative segment of the trajectory. The system does not appear to follow a limit cycle, and these preliminary results would suggest the presence of chaos in the D+S state, consistent with studies of chaos in periodically driven oscillators (d'Humieres et al., 1982). Indeed, if one looks at the two close points within the dashed circle, we observe how an initially small distance between them, rapidly grows in a few iterations steps, indicating a positive eigenvalue. For this simulation we have used a time-step *dt* = 0.001ms, to improve the resolution of the points in the plot. We have evaluated the time the neuron needs to circle the attractor, finding it to be of the order of ~ 5.3 spikes. Other drifting neurons take slightly longer or shorter. In Figure 6, we compute the fractal dimension of the here shown attractor.

Alternatively to a numerical evaluation of the Lyapunov exponents (a demanding task for large networks of spiking neurons), a somewhat more direct understanding of the chaotic state can be obtained by studying the relation between consecutive spike intervals. In Figure 5 we plot for this purpose a time series of 2000 consecutive interspike intervals [*s_i_*(*n*), *s_i_*(*n* + 1)] (corresponding to about 17s in real time), for one of the drifting neurons in the D+S state (with the parameters of the third example of Figure 1: *K* = 2.0 and Δ*K*/*K* = 0.2). We note that the spiking would be regular, viz *s_i_*(*n*) constant, for all neurons either in the fully drifting state (D) or in the fully synchronized state (S). The plot of consecutive spike intervals presented in Figure 5 can be viewed as a poor man's approximation to Takens' embedding theorem (Takens, 1981), which states that a chaotic attractor arising in a *d*-dimensional phase space (in our case *d* = 2*N*) can be reconstructed by the series of *d*-tuples of time events of a single variable.

With a blue line we follow in Figure 5 a representative segment of the trajectory, which jumps irregularly. A first indication that the attractor in Figure 5 may be chaotic comes from the observation that the trajectory does not seem to settle (within the observation window) within a limit cycle. The time series of consecutive spike-interval pairs will nevertheless approach any given previous pair [*s_i_*(*n*), *s_i_*(*n* + 1)] arbitrarily close, a consequence of the generic ergodicity of attracting sets (Gros, 2010). One of these close re-encounters occurs in Figure 5 near the center of the dashed circle, with the trajectory diverging again after the close re-encounter. This divergence indicates the presence of positive Lyapunov exponents.

**Figure 6:**
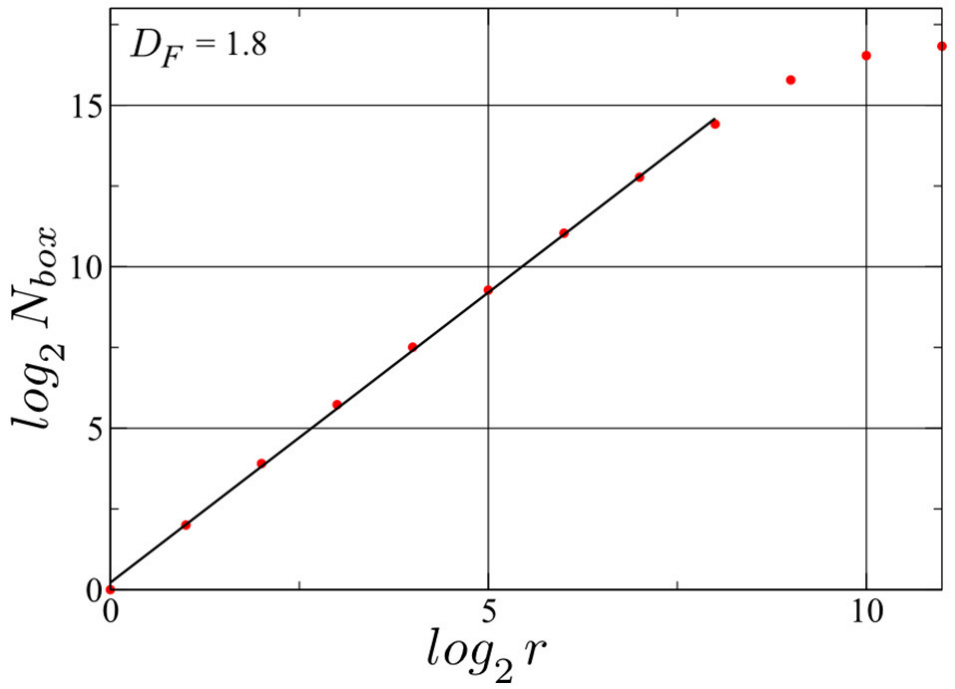
**Determination of the Minkowski (or box-counting) dimension for the attractor illustrated in Figure 5**. *N_box_* denotes the number of squares occupied with at least one point of the trajectory of consecutive pairs of spike intervals, when a grid of 2^*r*^ × 2^*r*^ squares is laid upon the attracting set shown in Figure 5. The fractal dimension *D_F_* = *log*(*N_box_*)/*log*(*r*) is then ~1.8. A time series with *N_spikes_* = 128000 spikes for the same drifting neuron in the same D+S state has been used. log_2_(*N_box_*) saturates at log_2_(*N_spikes_*) ≈ 16.97, observing that the linear range can be expanded further by increasing the number of spikes, albeit with an high cost in simulation time. Finally the resolution of the method is limited by the spike width.

We have determined the fractal dimension of the attracting set of pairs of spike intervals in the mixed phase by overlaying the attractor with a grid of 2^*r*^ × 2^*r*^ squares. For this calculation we employed a longer simulation with *N_spikes_* = 128, 000. The resulting box count, presented in Figure 6, yields a Minkowski or box-counting dimension *D_F_* ≈ 1.8, embeded in the 2D space of the plot, confirming such that the drifting neurons in the D+S phase spikes indeed chaotically. As a comparison, a limit cycle in this space, has a *D_F_* of 1. While we present here the result for one particular neuron, the same holds for every drifting neuron in this state, albeit with slightly different fractal dimension values. We note that the such determined fractal dimension is not the one of the full attractor in *d* = 2*N* phase space, for which tuples of 2*N* consecutive inter-spike intervals would need to be considered (Takens, 1981; Ding et al., 1993). Our point here is that a non-integer result for the single neuron *D_F_* strongly indicates that the full attractor (in the *d*-dimensional phase space) is chaotic.

We believe that the chaotic state arising in the mixed D+S state may be understood in analogy to the occurrence of chaos in the periodically driven pendulum (d'Humieres et al., 1982). A drifting neuron with a coupling constant *K* in the D+S does indeed receive two types of inputs to its conductance, compare Equation (4), with the first input being constant (resulting from the firing of the other drifting neurons) and with the second input being periodic. The frequency *r_syn_* of the periodic driving will then be strictly smaller than the natural frequency *r_K_* of the drifting neuron as resulting from the constant input (compare Figure 1). It is known from the theory of driven oscillators (d'Humieres et al., 1982) that the oscillator may not be able to synchronize with the external frequency, here *r_syn_*, when the frequency ratio *r_syn_*/*r_K_* is small enough and the relative strength of the driving not too strong.

#### 3.2.4. Robustness

In order to evaluate the robustness and the generality of the results here presented, we have evaluated the effects occurring when changing the size of the network and when allowing for variability in the connectivity matrix *w_ij_*. We have also considered an adiabatically increasing external input, as well as Gaussian noise.

**Figure 7:**
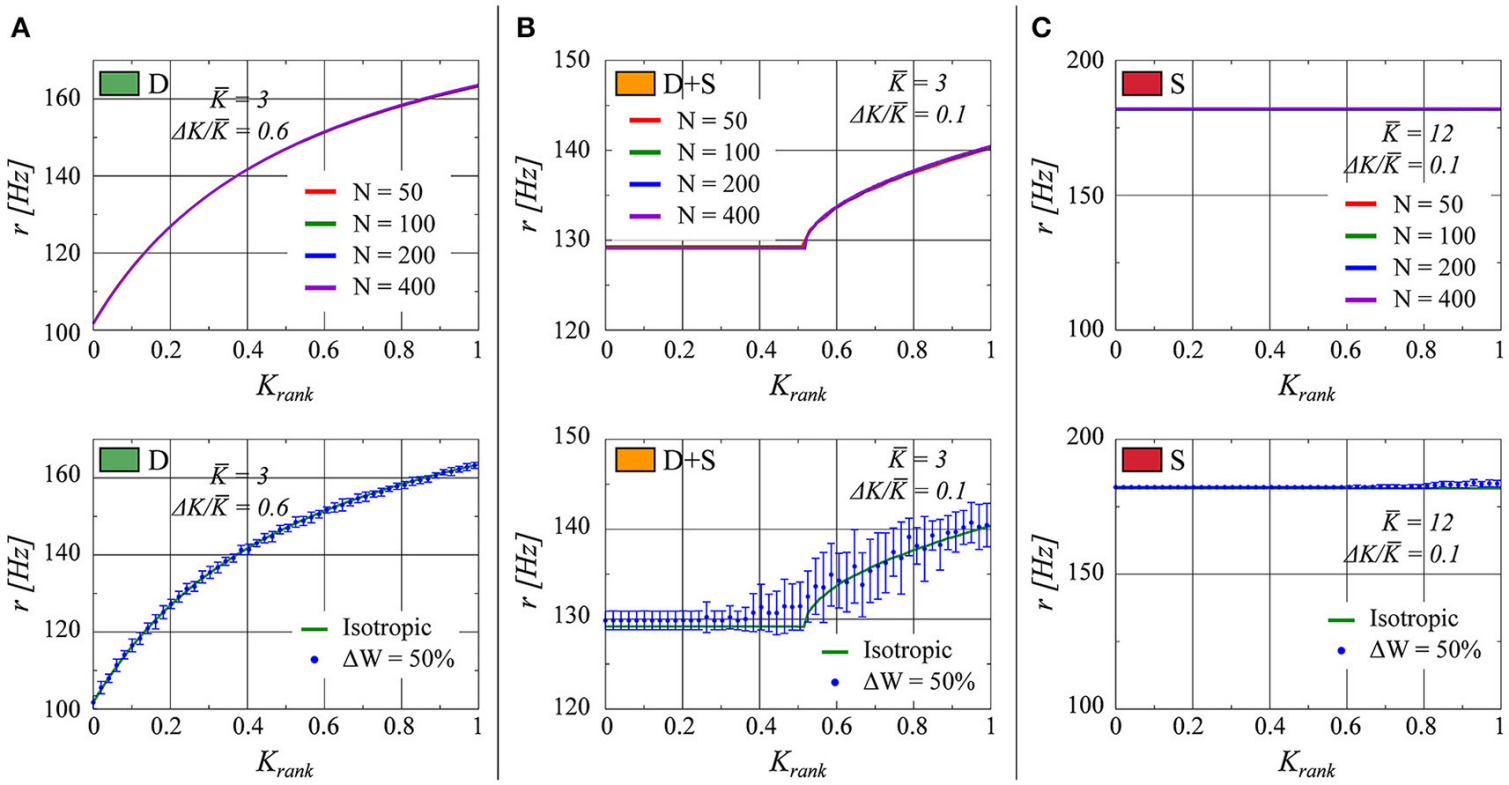
**As in Figure 1, the firing rate of each neuron in the network is presented as a function of the neuron's relative rank in K (from smaller to larger)**. **(A)** *K* = 3.0, Δ*K*/*K* = 0.6 (fully drifting: D). **(B)** *K* = 3.0, Δ*K*/*K* = 0.1 (partially drifting and synchronized: D+S). **(C)** *K* = 12.0, Δ*K*/*K* = 0.1 (fully synchronized: S). Top: Comparison for several network sizes *N*. Bottom: The numerical result for a network with a uniform connectivity matrix (red line) in comparison to a network in which the elements of the connectivity matrix are allowed to vary within 50% up or down from unity (Blue points. The error bars represent the standard deviation of twenty realizations of the weight matrix).

In Figure 7 (top half), the effect on the rate distribution of the network size is evaluated. Sizes of *N* = 50, 100, 200, and 400 have been employed. We observe that the plots overlap within the precision of the simulations. This result is on the one hand a consequence of the scaling *K_i_* ~ 1/*N* for the overall strength of the afferent links and, on the other hand, of the regularity in firing discussed in Section 3.2.2. The neural activity is driven by the mean field *r*(*t*) which is constant, in the thermodynamic limit *N* → ∞, in the fully drifting state and non-constant but smooth (apart from an initial jump) in the synchronized states. Fluctuations due to finite network sizes are already small for *N* ≈ 100, as employed for our simulations, justifying this choice.

In the previous sections, we considered the uniform connectivity matrix described by Equation (5). This allowed us to formulate the problem in terms of a mean-field coupling. We now analyze the robustness of the states found when a certain degree of variability is present in the weight matrix, viz when an extra variability term η is present:

(15) $$w_{ij}=1+\eta ,\;\eta \text{ random},\;i\neq j\;.$$

Here we consider η to be drawn from a flat distribution with zero mean and a width Δ*W*. Tests with Δ*W* = 10, 20, and 50% were performed. In Figure 7 (lower half), the results for Δ*W* = 50% are presented. We observe that the fully drifting state is the least affected by the variability in the weight matrix. On the other hand, the influence of variable weight matrices becomes more evident when the state is partially or fully synchronized, with the separation between the locked and the drifting neurons becoming less pronounced in the case of partial synchronization (lower panel of Figure 7B). The larger standard deviation evident for larger values of *K_rank_* in the lower panel of Figure 7C indicates the presence of drifting states in some of the ensemble realizations of weight matrices.

**Figure 8:**
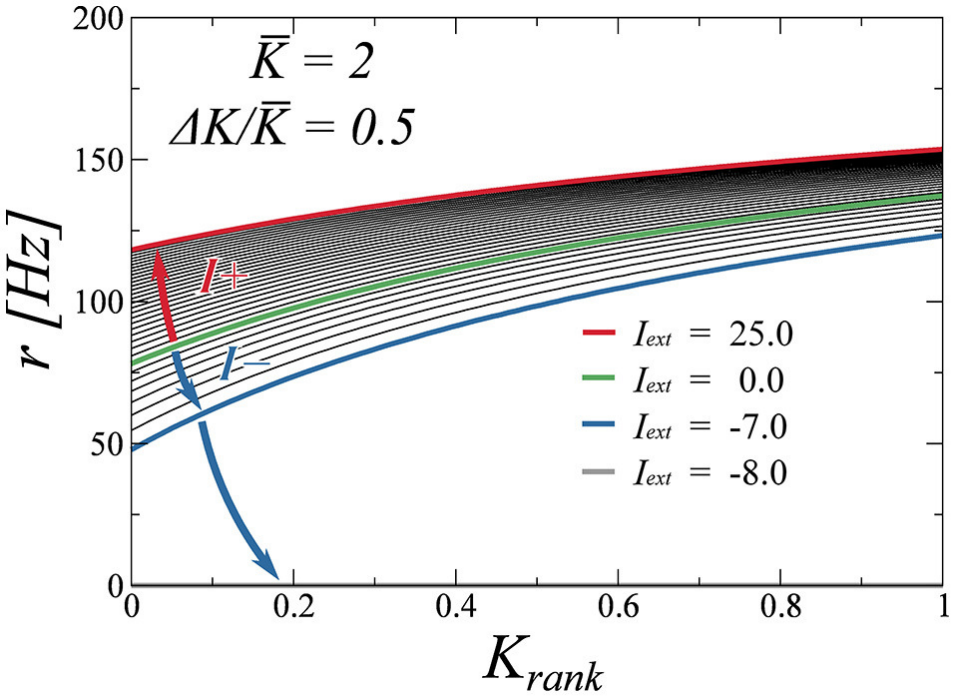
**Effect of an external input (either positive or negative), on the neural firing rates**. The input is either increased (blue arrow) or decreased (red arrow) from zero (green curve) adiabatically. The drifting state remains stable for a wide range of inputs, with the activity disappearing only for *I_ext_* < −7.0 (gray curve, coinciding with the *x*-axis).

Finally, we test the robustness of the drifting state when perturbed with an external stimulus. To determine the stability of the state, we adiabatically increase the external stimulus *I_ext_* and compute the firing rate as a function of the rank for several values of *I_ext_*. We do two excursions, one for positive values of *I_ext_* and another one for negative values. These plots are presented in Figure 8. We observe that the firing rates evolve in a continuous fashion, indicating that the drifting state is indeed stable. While positive inputs push the system to saturation, negative inputs reduce the average rate. We find, as is to be expected, that a large enough negative input makes the system silent. As a final test (not shown here), we have perturbed the system with random Gaussian uncorrelated noise, observing that the found attractors are all robust with respect to this type of noise as well.

## 4. Discussion

In the present work we have studied a network of excitable units, consisting exclusively of excitatory neurons. In absence of external stimulus, the network is only able to remain active through its own activity, in a self-organized fashion. Below a certain average coupling strength of the network the activity dies out, whereas, if the average coupling is strong enough, the excitable units will collectively behave as pulse-coupled oscillators.

We have shown how the variability of coupling strengths determines the synchronization characteristics of the network, ranging from fully asynchronous, to fully synchronous activity. Interestingly, this variability, together with the neurons' refractoriness, is enough to keep the neural activity from exploding.

While we have initially assumed a purely mean field coupling (by setting all the synaptic weights *w_ij_* = 1), only regulating the intensity with which a neuron integrates the mean field with the introduction of a scaling constant *K_i_*, we have later shown how the here found states also survive when we allow the *w_ij_s* to individually vary up or down by up to a 50% value. We have also shown how the variability in coupling strength makes the asynchronous or drifting state extremely robust with respect to strong homogeneous external inputs.

Finally, we have studied the time structure of spikes in the different dynamical states observed. It is in the time domain that we find the main difference with natural neural networks. Spiking in real neurons is usually irregular, and it is often modeled as Poissonian, whereas in our network we found a very high degree of regularity, even in the asynchronous state. Only in the partially synchronous state we found a higher degree of variability, as a result from chaotic behavior. We have determined the fractal dimension of the respective strange attractor in the space of pairs of consecutive interspike intervals, finding fractional values of roughly 1.8 for the different neurons in the state.

While it has been often stated that inhibition is a necessary condition for bounded and uncorrelated activity, we have show here that uncorrelated aperiodic (and even chaotic) activity can be obtained with a network of excitatory-only connections, in a stable fashion and without external input. We are of course aware that the firing rates obtained in our simulations are high compared to *in vivo* activity levels and that the degree of variability in the time domain of spikes is far from Poissonian. We have however incorporated in this work only variability of the inter-neural connectivity, keeping other neural properties (such as the neural intrinsic parameters) constant and homogeneous. In this sense, it would be interesting to study in future work, how intrinsic and synaptic plasticity (Triesch, 2007), modify these statistics, incorporating plasticity in terms of interspike-times (Clopath et al., 2010; Echeveste and Gros, 2015b), and in terms of neural rates (Bienenstock et al., 1982; Hyvärinen and Oja, 1998; Echeveste and Gros, 2015a). Here, instead of trying to reproduce the detailed connection statistics in the brain, which would in any case never be realistic without inhibitory neurons, we have shown how a minimal variability model in terms of non uniform link matrices is able to give rise to asynchronous spiking states, even without inhibition. Our results indicate therefore that further studies are needed for an improved understanding of which features of the asynchronous spiking state depend essentially on inhibition, and which do not.

We have shown here that autonomous activity (sustained even in the absence of external inputs) may arise in networks of coupled excitable units, viz for units which are not intrinsically oscillating. We have also proposed a new tool to study the appearance of chaos in spiking neural networks by applying a box counting method to consecutive pairs of inter-spike intervals from a single unit. This tool is readily applicable both to experimental data and to the results of theory simulations in general.

## Author contributions

RE carried out the simulations and produced the figures. CG guided the project and provided the theoretical framework. Both authors contributed to the writing of the article.

### Conflict of interest statement

The authors declare that the research was conducted in the absence of any commercial or financial relationships that could be construed as a potential conflict of interest.
